# Addressing the lack of research in rural communities through building rural health service research: Establishment of a research unit in Colac, a medium rural town

**DOI:** 10.1111/ajr.12860

**Published:** 2022-03-07

**Authors:** Laura Alston, Michael Field, Fiona Brew, Warren Payne, Drew Aras, Vincent L Versace

**Affiliations:** ^1^ Research Unit Colac Area Health Colac Victoria Australia; ^2^ Deakin Rural Health Faculty of Health Deakin University Geelong Victoria Australia; ^3^ The Global Obesity Centre Institute for Health Transformation Deakin University Warrnambool Victoria Australia; ^4^ Western Alliance Academic Health Science Centre Geelong Victoria Australia

Rural health services are leadership bodies within their communities and play a key role in addressing rural health disparities through providing evidence‐based prevention initiatives, community health care and clinical services.^1^ Relative to metropolitan settings, there is a paucity of rural health service research in Australia.^2^, ^3^ This has been theorised to contribute to a lack of rural‐specific scientific evidence, an important factor in the complex system driving health disparities in rural, regional and remote areas.^3^ For example, insufficient research evidence has contributed to a lack of solutions to address and prevent the persistent higher burden of non‐communicable diseases in rural areas, such as cardiovascular disease.^4^

Many challenges exist for publicly funded rural health services when undertaking research, including a lack of research funding, equipment or infrastructure, alongside workforce size, retention and supply (relative to need) issues.^3^ A recent study by Alston et al. found that a lack of rural‐specific evidence, and rural health service research, made it more difficult for policy‐makers to make evidence‐informed decisions to improve the health of rural communities.^4^ Policy‐makers at all government levels felt there was a responsibility for rural health services to generate research evidence, despite minimal government investment, infrastructure and workforce to support such efforts.^4^ Exploration is needed to understand how rural health services can build research programs and further address important gaps in the rural health literature. This will in part address health knowledge gaps for current and future rural and regional dwelling generations. Although there is some discussion in the literature around how embedded research should work in the Australian rural health service setting, published case studies, success stories and empirical research showing implementation are sparse.^3^, ^5^, ^6^

In this *AJRH Practice Piece*, we describe a success story of building health service research capacity and capability and discuss key learnings from Colac Area Health. Not only Colac is classified as a ‘medium rural towns’ (MM4) by the Modified Monash Model, but also services surrounding regions are classified as MM5 or ‘small rural towns’ (total population >21 000).^7^, ^8^ The postcode that covers Colac ranks at the lower end of the Socio‐Economic Indexes for Areas (SEIFA).^9^ It is classified as Decile 1 by the Index of Education and Occupation (IEO), and Decile 2 by the Index of Relative Socio‐Economic Advantage and Disadvantage (IRSAD), the Index of Relative Socio‐Economic Disadvantage (IRSD) and the Index of Economic Resources (IER).^9^ This is consistent with other MM4 areas in Victoria where most of the population reside in IRSAD Deciles 1–3.^10^ Colac Area Health is the largest health service in the Colac Otway region and provides services across acute, aged care and community health for more than 21 000 rural Victorians.^11^ Relative to metropolitan areas, the catchment community has increased rates of obesity, cardiovascular disease, mental health conditions, and higher sugar‐sweetened beverage consumption and suboptimal diets, ultimately leading to increased disease burden on the health service.^12^ In 2019/2020 financial year, Colac Area Health provided community health services to 6541 rural residents, as well as provided 6530 acute care separations, and managed 10 592 presentations to urgent care.^11^ In 2020, there were 241 full‐time equivalent clinical staff (nursing, medical and allied health) and two research‐specific roles of 0.7FTE. The health service plays a pivotal leadership role in the region and is an economic driver employing many local people. Although the health service had been a partnering site on university‐driven research projects, essentially no internally led research was undertaken at the health service prior to 2018, and the health service had never received research‐specific funding. This had come to the awareness of Executive and Board staff as a service and practice gap for Colac Area Health, as well as a lost opportunity for staff professional development. The health service's leadership understood that advancing research capacity and capability would be new and potentially challenging for the organisation, as it was not part of the current culture or embedded into existing roles, not unusual for health services of this size. This, alongside historical funding, infrastructure and workforce deficiencies inhibited the enabling environment required to undertake research across rural health services more broadly.

In 2018, the situation changed, augmented by the wider policy environment and advocacy from rural health groups such as the Spinifex Network.^2^ With competitive seed funding from the Western Alliance Academic Health Science Centre (Western Alliance), of which Colac Area Health is a member, for a clinical malnutrition project, championed by allied health staff and an internally employed clinical who was a PhD student at the time^13^ (in collaboration with Deakin University), the first‐ever health service‐driven research project was initiated. The internally led project provided new employment opportunities for Colac Area Health staff and led to increased research skill development among >15 staff members, as well as increased networking with local researchers. This project helped to ‘normalise’ research as part of usual business, along with support from the leadership in allowing some of the clinicians to contribute to the research as part of their current role (in addition to the funded research assistant position). With this success, a paper was published,^13^ resulting in a sense of achievement among the staff and a state‐level Allied Health Award. The study led to immediate improvements in policy, practice and education at the health service^11^ and progressed the normalisation of the internal research culture. The direct improvements included an increase in awareness of the condition of focus, a newly designed and evidence‐informed education program for clinical staff and revised policy to include better screening and identification practices. This initial study, along with leadership and collaboration, has since led to further expansion of research activity through engaging and supporting Colac Area Health staff in clinician‐led research. The leadership encouraged the internal staff involved in the project to continue research. They were supported to continue applying for more research grants, and this coincided with more funding from the Western Alliance in the form of a specific research translation support role. There was also further relationship building and investment from the local UDRH (Deakin Rural Health) for the internal researcher based within the health service. Following on from the completion of the first internally led project, the Colac Area Health Executive team officially opened the research unit in 2020, with further investment from Deakin Rural Health and the Western Alliance. The Deakin Rural Health had already established collaborations with larger regional health services in the region and included direct support from four senior research staff. In consultation with rural health services, the Western Alliance co‐designed positions specifically aimed at building research capacity in rural health services and provided a research translation officer to support research, research translation and research capacity building at Colac Area Health, and across the surrounding region.

Hulcombe et al.^14^ outline a framework for building research capacity in the Australian public health context, and our case study reflects major components of the framework including ‘Leadership and collaboration’, ‘Support for our researchers’ and ‘Translating evidence into practice’ (summarised in Figure 1).

**FIGURE 1:**
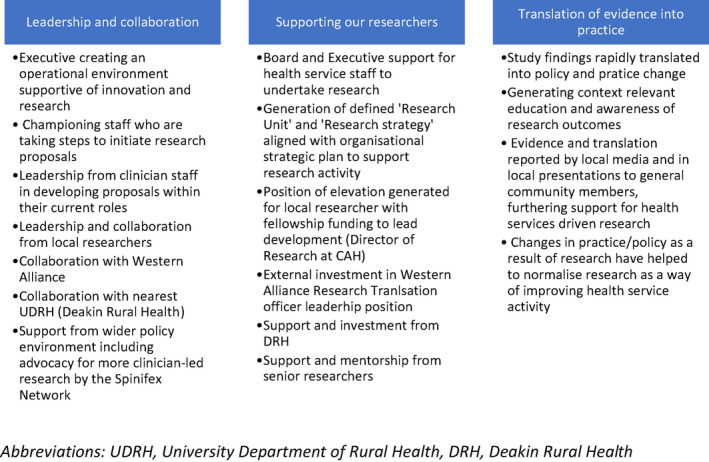
Present case study summarised by the domains of Hulcombe et al's approach to building research capacity for health practitioners in a public health environment

Since the first seed grant, five Colac Area Health staff members have successfully led their first research project, supported by the research unit, and these projects have led to direct improvements in the care of patients at the health service, peer‐reviewed publications, and contributed to further research grant success for Colac Area Health. Overall, the research unit has directly engaged at over 15 staff and influenced practice among many more through localised dissemination efforts. Importantly, the evidence generated by the health service is being translated into practice. The health service is currently a leader in or partnering site (with named investigators on the submission) on more than 30 projects, spanning topics such as nutrition, prevention of chronic diseases, aged care, surgery, maternity services and impact of COVID‐19.

Our case study shows that the initiation of an internally led project, alongside executive, Deakin University, and external participation in and support from an academic health science centre can lead to further expansion of research within rural health services. This case study also reflects Hulcombe et al's^14^ current Australian contextual framework within the literature. Success stories from small scale, yet achievable, research projects can influence existing cultures and assist with normalising research, empowering staff, improving skills and reducing fear of research among rural health service staff. The growing research reputation of the health service will also make it an attractive site for the ‘rural arm’ of future studies.

There will certainly be ongoing threats to the sustainability of the research unit, mainly funding issues, as the health service does not receive research‐specific funding within the current service model. The model is currently sustained by a small investment from the Health Service, the UDRH, the Academic Health Science Centre and other competitive funding. To assist with reducing the ongoing funding challenges that the research unit will face the health service executives are currently drafting a formal memorandum of understanding with the supporting University Department of Rural Health. Potential generalisability of this model is limited by there being existing motivation among some staff members (or existing research capacity), including a clinician who was also undertaking a PhD and had the skills to apply for the original grant application. The PhD student had already been engaged with the Western Alliance, who had previously contributed to the training and development of the PhD candidate. This highlights the importance of collaboration between Academic Health Science Centre's in regional areas, and the important role they play in developing clinical health service staff in research. Another challenge is that current research staff may eventually leave the organisation to pursue university‐based careers, and this is currently being mitigated by the research unit (and collaborators) to focus on developing and supporting local clinicians, as well as attracting PhD students to undertake health service research in the region. The aim of this is to embed a research culture and ensure sustained capacity within the health service.

This case study has led to reflections across the health service and surrounding region around the potential of research in rural health services of all sizes. Further research in this area should include economic analyses of this model (and others) to determine cost‐effectiveness alongside the impacts rural health service research unit has on employees and the health of the wider rural community. There is a case for large‐scale research funding for rural health services, to build capacity and capability, normalise research cultures and improve the availability of rural‐specific research evidence. This will play a role in addressing and overcoming the persistent rural health inequalities for future rural Australian generations.

## ETHICS STATEMENT

None.

## CONFLICT OF INTEREST

There is no conflict of interest to declare.

## AUTHOR CONTRIBUTIONS

LA: conceptualization; formal analysis; investigation; methodology; project administration; resources; supervision; writing – original draft; writing – review & editing. MF: conceptualization; data curation; investigation; methodology; project administration; writing – original draft; writing – review & editing. FB: conceptualization; investigation; methodology; project administration; resources; supervision; writing – original draft; writing – review & editing. WP: conceptualization; methodology; project administration; resources; supervision; writing – original draft; writing – review & editing. DA: conceptualization; investigation; methodology; project administration; resources; writing – original draft; writing – review & editing. VLV: conceptualization; methodology; project administration; resources; supervision; writing – original draft; writing – review & editing.
